# Subtype of atrial fibrillation and the outcome of transcatheter aortic valve replacement: The FinnValve Study

**DOI:** 10.1371/journal.pone.0238953

**Published:** 2020-09-11

**Authors:** Jussi Jaakkola, Samuli Jaakkola, K. E. Juhani Airaksinen, Annastiina Husso, Tatu Juvonen, Mika Laine, Marko Virtanen, Pasi Maaranen, Matti Niemelä, Timo Mäkikallio, Mikko Savontaus, Tuomas Tauriainen, Antti Valtola, Antti Vento, Markku Eskola, Peter Raivio, Fausto Biancari

**Affiliations:** 1 Heart Center, Turku University Hospital and University of Turku, Turku, Finland; 2 Heart Center, Kuopio University Hospital, Kuopio, Finland; 3 Heart and Lung Center, Helsinki University Hospital, Helsinki, Finland; 4 Research Unit of Surgery, Anesthesiology and Critical Care, University of Oulu, Oulu, Finland; 5 Heart Hospital, Tampere University Hospital and Faculty of Medicine and Health Technology, Tampere University, Tampere, Finland; 6 Department of Internal Medicine, Oulu University Hospital, Oulu, Finland; Erasmus Medical Center, NETHERLANDS

## Abstract

Whether the subtype of atrial fibrillation affects outcomes after transcatheter aortic valve replacement for aortic stenosis is unclear. The nationwide FinnValve registry included 2130 patients who underwent primary after transcatheter aortic valve replacement for aortic stenosis during 2008–2017. Altogether, 281 (13.2%) patients had pre-existing paroxysmal atrial fibrillation, 651 (30.6%) had pre-existing non-paroxysmal atrial fibrillation and 160 (7.5%) were diagnosed with new-onset atrial fibrillation during the index hospitalization. The median follow-up was 2.4 (interquartile range: 1.6–3.8) years. Paroxysmal atrial fibrillation did not affect 30-day or overall mortality (p-values >0.05). Non-paroxysmal atrial fibrillation demonstrated an increased risk of overall mortality (hazard ratio: 1.61, 95% confidence interval: 1.35–1.92; p<0.001), but not 30-day mortality (p = 0.084). New-onset atrial fibrillation demonstrated significantly increased 30-day mortality (hazard ratio: 2.76, 95% confidence interval: 1.25–6.09; p = 0.010) and overall mortality (hazard ratio: 1.68, 95% confidence interval: 1.29–2.19; p<0.001). The incidence of early or late stroke did not differ between atrial fibrillation subtypes (p-values >0.05). In conclusion, non-paroxysmal atrial fibrillation and new-onset atrial fibrillation are associated with increased mortality after transcatheter aortic valve replacement for aortic stenosis, whereas paroxysmal atrial fibrillation has no effect on mortality. These findings suggest that non-paroxysmal atrial fibrillation rather than paroxysmal atrial fibrillation may be associated with structural cardiac damage which is of prognostic significance in patients with aortic stenosis undergoing transcatheter aortic valve replacement.

## Introduction

Transcatheter aortic valve replacement (TAVR) has demonstrated its efficacy in the treatment of severe symptomatic aortic stenosis (AS) among patients who are inoperable or at a high or moderate surgical risk [1, 2]. Atrial fibrillation (AF) and AS share multiple important risk factors, and AS causes a chronic increase in left ventricular filling pressure, which further predisposes to AF. Indeed, AF is an exceedingly common condition in the presence of AS with a 16–51% prevalence among patients scheduled for TAVR [3]. Furthermore, new-onset AF is a common finding during the immediate postoperative period after TAVR with the incidence ranging from 6 to 35% [4–6]. AF carries important prognostic implications in patients with AS, and the adverse effect of pre-existing AF on mortality in patients undergoing TAVR is well-established [3, 5–10]. However, little is known about whether mortality outcomes differ between patients with paroxysmal or permanent AF and there is conflicting evidence on the prognostic effect of new-onset AF after TAVR [7–9]. Moreover, stroke is an important adverse event after TAVR affecting up to 5% of patients scheduled for the procedure, while the effect of AF on stroke incidence in this population is currently unclear [7, 11]. The aim of the current study was to assess the effect of the subtype of pre-existing AF on outcomes after TAVR for AS and to explore the influence of new-onset AF on mortality in the multicenter FinnValve registry.

## Material and methods

### Study population

The FinnValve registry is a Finnish multicenter nationwide registry including retrospectively collected data from consecutive patients undergoing TAVR or surgical aortic valve replacement with a bioprosthesis for severe AS at all five Finnish university hospitals (Turku, Helsinki, Kuopio, Tampere and Oulu) between January 2008 and November 2017 (Clinical trial registration: ClinicalTrials.gov Identifier: NCT03385915) [12]. The study protocol was approved by the Institutional Review Boards of all participating centers and the Ethics Committee of the National Institute for Health and Welfare. Informed consent was waived due to the retrospective registry nature of the study. The study conforms to the Declaration of Helsinki as revised in 2002.

All patients over the age of 18 years with AS undergoing TAVR or surgical aortic valve replacement with a bioprosthesis during the study period meeting the inclusion criteria were included in the registry [12]. The exclusion criteria of the registry were previous TAVR or surgical aortic valve intervention, concomitant major cardiac procedure involving the ascending aorta or other heart valves or structures, surgical or transcatheter procedure for isolated aortic valve regurgitation and acute endocarditis. Finally, 2130 patients undergoing TAVR and 4333 patients undergoing surgical aortic valve replacement were included in the registry. The current analysis focused only on patients on whom TAVR was performed.

The diagnosis and subtype of atrial fibrillation was determined from the clinical records of the study patients undergoing TAVR. Patients were classified in to four categories according to presence and subtype of AF: pre-existing paroxysmal AF, pre-existing non-paroxysmal AF, new-onset AF and no AF. Non-paroxysmal AF included chronic and persistent AF. New-onset AF was defined as AF diagnosed during the index hospitalization at the operative center. Among the patients undergoing TAVR, 281 (13.2%) patients had pre-existing paroxysmal AF, 651 (30.6%) patients had pre-existing non-paroxysmal AF and 160 (7.2%) patients were diagnosed with new-onset AF, while 1038 (48.7%) patients had no history of AF at the time of hospital discharge.

### Study protocol

Data on baseline and operative characteristics and postoperative outcomes were retrospectively collected in a standardized online electronic data collection form. Data on the date of death were obtained from the Population Register Center, a governmental agency, which reviews all death certificates issued in Finland. Data on cardiovascular interventions were obtained from the Finnish National Institute for Health and Welfare registry, which collects information on the diagnosis and treatment of patients admitted to any local, regional or tertiary hospital. The last day of follow-up was January 26^th^, 2019. Follow-up was considered complete for all patients except for those not residing in Finland, and their follow-up was truncated at hospital discharge.

### Definition criteria

Baseline variables were defined according to the EuroSCORE II criteria [13]. Coronary artery disease was defined as stenosis of ≥50% of any of the main coronary arteries. Severe frailty was defined as Geriatric Status Scale (GSS) 2–3 [14]. Pulmonary disease was defined as long-term use of bronchodilators or steroids for lung disease. Extracardiac arteriopathy was defined as one or more of the following: claudication, carotid occlusion or >50% stenosis, amputation for arterial disease and previous or planned intervention on the abdominal aorta, limb arteries or carotid arteries. Poor mobility was defined as severe impairment of mobility due to musculoskeletal or neurological dysfunction. The patients were defined as having a critical preoperative state if one or more of the following were present preoperatively: ventricular tachycardia or ventricular fibrillation or aborted sudden death, preoperative cardiac massage, preoperative ventilation before anesthetic room, preoperative inotropes or intra-aortic balloon pump and preoperative acute renal failure. Anemia was defined according to the World Health Organization criteria as hemoglobin <12 g/dL in women and <13 g/dL in men. Life-threatening or major bleeding was defined according to the Valve Academic Research Consortium 2 criteria [15]. All TAVR devices were classified as older or newer generation prostheses. CoreValve (Medtronic, Minneapolis, MN, USA), Engager (Medtronic, Minneapolis, MN, USA), Portico (St. Jude Medical, Minneapolis, MN, USA) Sapien (Edwards Lifesciences, Irvine, CA, USA) and Sapien XT (Edwards Lifesciences, Irvine, CA, USA) were considered older generation prostheses. Lotus (Boston Scientific, Marlborough, MA, USA), Lotus Edge (Boston Scientific, Marlborough, MA, USA), Evolut (Medtronic, Minneapolis, MN, USA), Evolut R (Medtronic, Minneapolis, MN, USA), Evolut Pro (Medtronic, Minneapolis, MN, USA), Sapien 3 (Edwards Lifesciences, Irvine, CA, USA) and Acurate Neo (Boston Scientific, Marlborough, MA, USA) were considered newer generation prostheses.

### Outcome measures

The primary outcome measure of the current study was all-cause overall mortality. The secondary outcome measures were all-cause 30-day mortality and 5-year mortality, early stroke occurring during the index hospitalization, late stroke occurring after discharge as well as combined early or late stroke. Outcome analyses were not landmarked.

### Statistical analysis

Statistical analyses were performed with the IBM SPSS Statistics software (version 26.0, SPSS, Inc., Chicago, Illinois) and R software (version 4.0.2, https://www.R-project.org). The normality assumption of continuous variables was assessed visually and with the Kolmogorov-Smirnov test of normality. Continuous data are presented as median (interquartile range) and categorical variables as absolute number and percentage. The Fisher’s exact test and the chi-square test were used to compare differences between proportions, and the Kruskal-Wallis test and the Mann-Whitney U-test to analyze continuous variables. The Kaplan-Meier method was used to assess survival. Differences in survival between patients with paroxysmal, non-paroxysmal or new-onset AF and those without AF were evaluated with the log-rank test. A stepwise Cox proportional-hazards model with the Wald backward method was used to determine the adjusted hazard of overall, 30-day and 5-year mortality mortality associated with AF. All variables with p<0.1 in univariate Cox regression analyses were included in to the multiple Cox model. The proportional hazards assumption was ensured by assessing the test based on Schoenfeld residuals, as well as by graphical assessment of the ln-ln plot of the survival curves. Binary logistic regression models with the Wald backward method were used to define the adjusted odds ratio of early, late or any stroke during the follow-up. A binary logistic regression analysis with the Wald backward method was also performed to assess the independent predictors of new-onset AF. The goodness of fit of the binary logistic regression models were estimated by the Hosmer-Lemeshow's test. All variables with p<0.1 in univariate analyses were included in to the logistic regression models. Two-sided differences were considered significant if the null hypothesis could be rejected at the 0.05 probability level.

## Results

The median age of the patients was 82.4 (78.0–85.8) years and 958 (45.0%) were men. The baseline and operative characteristics of the patients are presented in Table 1.

**Table 1:** Baseline and operative characteristics.

Covariates	No AF n = 1038	Paroxysmal AF n = 281	Non-paroxysmal AF n = 651	New-onset AF n = 160	P value
Age, years	82.1 (77.9–85.7)	82.5 (77.1–85.7)	82.6 (78.8–86.2)	82.6 (78.2–85.6)	0.131
Male sex	453 (43.6)	115 (40.9)	318 (48.8)	72 (45.0)	0.088
Diabetes	266 (25.6)	79 (28.1)	226 (34.7)	34 (21.3)	<0.001
Coronary artery disease	292 (28.1)	75 (26.7)	181 (27.8)	55 (34.4)	0.339
History of myocardial infarction	134 (12.9)	52 (18.5)	94 (14.4)	25 (15.6)	0.115
Recent myocardial infarction	27 (2.6)	10 (3.6)	9 (1.4)	3 (1.9)	0.174
Extracardiac arteriopathy	205 (19.7)	46 (16.4)	126 (19.4)	35 (21.9)	0.501
History of stroke or TIA	167 (16.1)	61 (21.7)	128 (19.7)	28 (17.5)	0.093
Pulmonary disease	209 (20.1)	71 (25.3)	138 (21.2)	38 (23.8)	0.259
Permanent pacemaker	62 (6.0)	41 (14.6)	109 (16.7)	4 (2.5)	<0.001
Active malignancy	39 (3.8)	11 (3.9)	26 (4.0)	8 (5.0)	0.903
Acute heart failure within 90 days	105 (10.1)	52 (18.5)	125 (19.2)	26 (16.3)	<0.001
Previous cardiac surgery	201 (19.4)	42 (14.9)	160 (24.6)	28 (17.5)	0.003
Poor mobility	82 (7.9)	23 (8.2)	82 (12.6)	19 (11.9)	0.008
Severe frailty	136 (13.1)	40 (14.2)	114 (17.5)	28 (17.5)	0.069
eGFR, mL/min/1.73 m^2^	66.4 (52.9–82.0)	60.5 (45.4–75.8)	58.7 (43.8–76.3)	67.3 (54.5–84.8)	<0.001
eGFR <30 mL/min/1.73 m^2)^	32 (3.1)	15 (5.3)	29 (4.5)	7 (4.4)	0.258
BMI, kg/m^2^	26.6 (23.8–30.1)	26.9 (24.0–29.7)	26.4 (23.9–30.1)	26.0 (23.7–29.3)	0.693
Anemia	463 (44.6)	151 (53.7)	300 (46.1)	81 (50.6)	0.038
CHA_2_DS_2_-VASc score	4 (3–5)	4 (3–5)	4 (4–5)	5 (4–5)	<0.001
Preoperative antithrombotic therapy					<0.001
None	177 (17.1)	12 (4.3)	12 (1.8)	23 (14.4)	
AC	56 (5.4)	161 (57.3)	520 (79.9)	16 (10.0)	
AC and SAPT	43 (4.1)	52 (18.5)	76 (11.7)	8 (5.0)	
AC and DAPT	9 (0.9)	14 (5.0)	22 (3.4)	4 (2.5)	
SAPT	621 (59.8)	30 (10.7)	17 (2.6)	89(55.6)	
DAPT	132 (12.7)	12 (4.3)	4 (0.6)	20 (12.5)	
Dyspnea, NYHA					<0.001
Class I	14 (1.3)	3 (1.1)	0 (0.0)	0 (0.0)	
Class II	212 (20.4)	42 (14.9)	68 (10.4)	24 (15.0)	
Class III	700 (67.4)	199 (70.8)	501 (77.0)	123 (76.9)	
Class IV	112 (10.8)	37 (13.2)	82 (12.6)	13 (8.1)	
Combined aortic valve stenosis and regurgitation	344 (32.2)	106 (37.7)	183 (28.1)	43 (26.9)	0.016
Aortic valve maximum gradient, mmHg	78 (65–94)	75 (63–85)	72 (59–85)	76 (65–93)	<0.001
Aortic valve mean gradient, mmHg	48 (40–59)	45 (38–54)	43 (35–53)	47 (39–60)	<0.001
Recent aortic valve balloon valvuloplasty	17 (1.6)	15 (5.3)	25 (3.8)	8 (5.0)	0.001
Left ventricle ejection fraction					<0.001
>50%	788 (76.1)	207 (73.7)	421 (64.8)	114 (71.3)	
31–50%	213 (20.6)	66 (23.5)	192 (29.5)	35 (21.9)	
21–30%	24 (2.3)	8 (2.8)	29 (4.5)	11 (6.9)	
<21%	10 (1.0)	0 (0.0)	8 (1.2)	0 (0.0)	
Systolic pulmonary artery pressure*					<0.001
<31 mmHg	396 (46.4)	84 (35.9)	132 (22.8)	52 (37.7)	
31–55 mmHg	359 (42.1)	113 (48.3)	320 (55.4)	61 (44.2)	
>55 mmHg	98 (11.5)	37 (15.8)	126 (21.8)	25 (18.1)	
STS score, %	3.4 (2.5–5.0)	4.2 (2.9–6.1)	4.1 (2.9–5.7)	3.8 (2.6–5.5)	<0.001
EuroSCORE II, %	4.5 (2.6–7.8)	4.5 (2.8–8.3)	6.0 (3.6–10.3)	5.1 (3.0–8.7)	<0.001
Urgent or emergency procedure	68 (6.6)	29 (10.3)	49 (7.5)	12 (7.5)	0.204
Critical preoperative state	22 (2.1)	5 (1.8)	13 (2.0)	8 (5.0)	0.109
Non-transfemoral access	100 (9.6)	36 (12.8)	100 (15.4)	51 (31.9)	<0.001
Transapical access	72 (6.9)	24 (8.5)	65 (10.0)	34 (21.3)	<0.001
Prosthesis type					<0.001
Self-expandable	240 (23.3)	55 (19.7)	129 (19.9)	15 (9.4)	
Balloon-expandable	675 (65.5)	183 (65.6)	461 (71.1)	125 (78.6)	
Mechanically expanded	116 (11.3)	41 (14.7)	58 (9.0)	19 (11.9)	
Newer generation prosthesis	733 (70.7)	195 (69.6)	416 (63.9)	90 (56.3)	<0.001
Balloon post-dilatation	34 (3.3)	8 (2.8)	27 (4.1)	8 (5.0)	0.526
Postop. moderate or severe paravalvular regurgitation	35 (3.4)	5 (1.8)	31 (4.8)	8 (5.0)	0.111
Length of hospital stay, days	2 (4–6)	3 (5–7)	3 (5–7)	7 (4–12)	<0.001
Antithrombotic therapy at discharge					<0.001
None	18 (7.7)	11 (3.9)	19 (2.9)	12 (7.5)	
AC	154 (14.8)	175 (62.3)	473 (72.7)	58 (36.3)	
AC and SAPT	79 (7.6)	48 (17.1)	127 (19.5)	37 (23.1)	
AC and DAPT	32 (3.1)	10 (3.6)	20 (3.1)	11 (6.9)	
SAPT	107 (10.3)	16 (5.7)	5 (0.8)	5 (3.1)	
DAPT	648 (62.4)	21 (7.5)	7 (1.1)	37 (23.1)	

Continuous variables are reported as median (interquartile range); categorical variables are reported as counts and percentages.

*Data missing on 327 patients.

Abbreviations: AC, anticoagulant; AF, atrial fibrillation; CHA_2_DS_2_-VASc, congestive heart failure, hypertension, age >74 years, diabetes, history of stroke or transient ischemic attack, vascular disease, age 65–74 years, sex category (female); DAPT, double antiplatelet therapy; eGFR, estimated glomerular filtration rate according to the MDRD equation; MDRD, Modification of Diet in Renal Disease; NYHA, New York Heart Association; SAPT, single antiplatelet therapy; STS, Society of Thoracic Surgery; TIA, transient ischemic attack.

**Fig 1:**
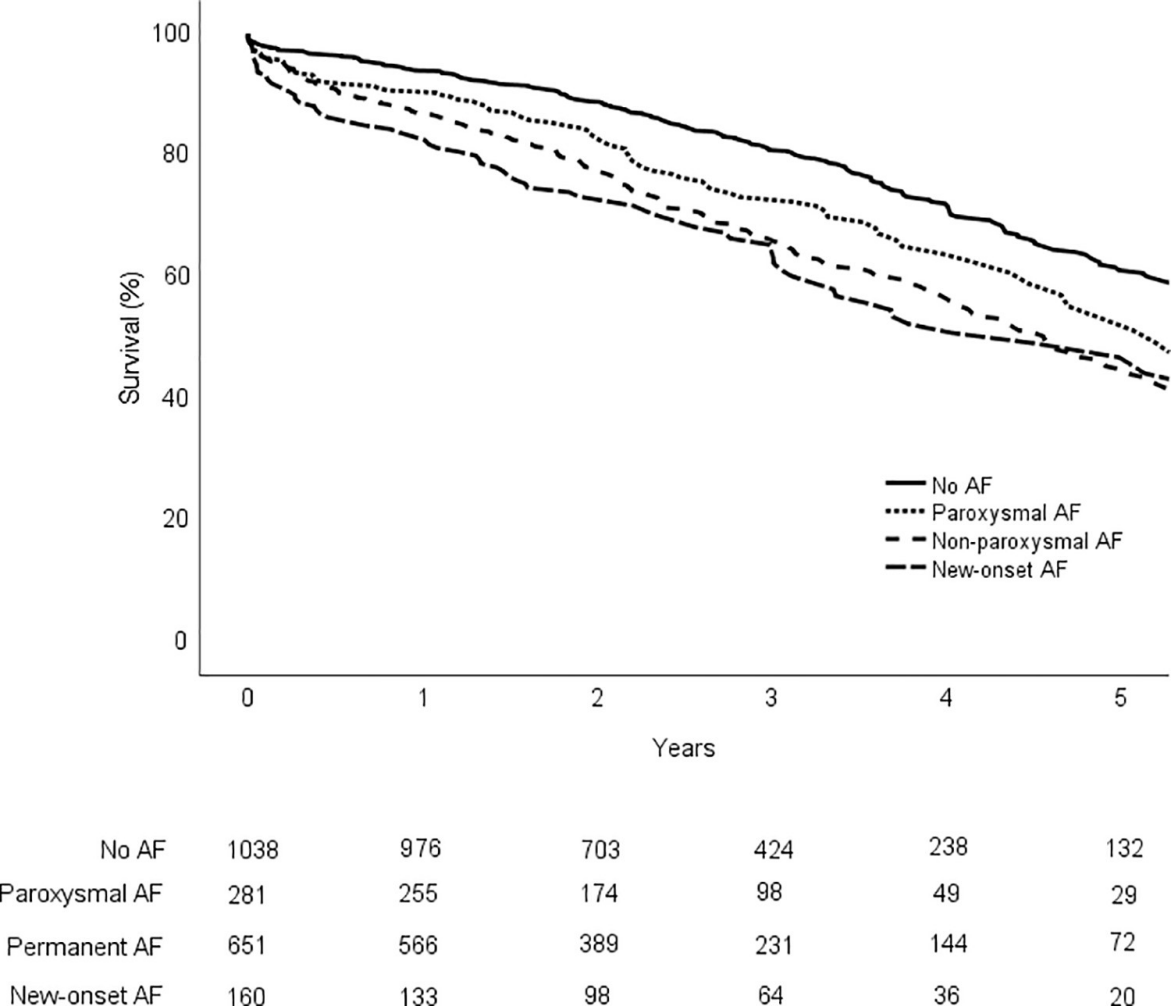
Kaplan-Meier estimate of survival in patients with paroxysmal, non-paroxysmal, new-onset or no AF undergoing transcatheter aortic valve replacement. Number at the bottom of graph are patients at risk.

During the follow-up (median 2.4 (1.6–3.89 years), the primary outcome measure of all-cause mortality occurred in 85 (30.2%) patients with paroxysmal AF, 265 (40.7%) patients with non-paroxysmal AF and 71 (44.4%) patients with new-onset AF. The median survival time was 4.6 (2.2–7.0) years in patients with non-paroxysmal AF, 5.2 (2.7–9.0) years in patients with paroxysmal AF, 4.5 (3.2–5.1) years in patients with new-onset AF and 6.6 (3.6–8.6) years in those with no AF (log-rank <0.001). The hazard ratio (HR) of all-cause mortality was 1.36 (95% confidence interval (CI): 1.06–1.74; p = 0.014) for paroxysmal AF, 1.81 (95% CI: 1.52–2.15; p<0.001) for non-paroxysmal AF and 1.84 (95% CI: 1.1.42–2.39; p<0.001) for new-onset AF as compared with the 259 (25.0%) patients without AF. Survival in patients with paroxysmal, non-paroxysmal, new-onset and no AF before TAVR are presented in Fig 1.

The adjusted risk estimates of study outcomes are shown in Table 2. After adjusting for baseline and operative covariates, non-paroxysmal and new-onset AF were associated with increased all-cause mortality compared to no AF, whereas paroxysmal AF was not. Compared to pre-existing non-paroxysmal AF, lesser hazards of overall mortality were observed in patients with no AF (HR: 0.62, 95% CI: 0.52–0.74, p<0.001) and pre-existing paroxysmal AF (HR: 0.76, 95% CI: 0.59–0.97, p = 0.029), whereas similar hazard was observed in patients with new-onset AF (HR: 0.89, 95% CI: 0.68–1.16, p = 0.387). Higher odds of 30-day mortality was observed in those with new-onset AF, while those with paroxysmal or non-paroxysmal AF demonstrated no difference in contrast to patients with no AF (Table 2). No difference in the adjusted odds of early or late stroke between those with no AF, paroxysmal AF, non-paroxysmal AF or new-onset AF was observed (Table 2).

**Table 2:** Adjusted risk estimates of study outcomes*.

	No AF	Paroxysmal AF			Non-paroxysmal AF			New-onset AF		
	N = 1038	N = 281	Risk estimate (95% CI)	P value	N = 651	Risk estimate (95% CI)	P value	N = 160	Risk estimate (95% CI)	P value
Overall mortality	259 (25.0)	85 (30.2)	1.22 (0.95–1.60)	0.119	265 (40.7)	1.61 (1.35–1.92)	<0.001	71 (44.4)	1.43 (1.09–1.89)	0.010
30-day mortality	19 (1.8)	10 (3.6)	1.79 (0.83–3.19)	0.136	23 (3.5)	1.73 (0.94–3.19)	0.077	10 (6.3)	2.76 (1.25–6.09)	0.012
5-year mortality	228 (22.0)	78 (27.8)	1.23 (0.94–1.60)	0.126	239 (36.7)	1.60 (1.33–1.93)	<0.001	66 (41.3)	1.49 (1.12–1.99)	0.006
Early stroke	24 (2.3)	8 (2.8)	1.15 (0.51–2.61)	0.738	14 (2.2)	0.90 (0.46–1.74)	0.730	8 (5.0)	2.09 (0.92–4.76)	0.080
Late stroke	38 (3.7)	14 (5.0)	1.35 (0.72–2.54)	0.352	31 (4.8)	1.19 (0.73–1.95)	0.480	8 (5.0)	1.22 (0.55–2.69)	0.621
Early or late stroke	60 (5.8)	20 (7.1)	1.21 (0.71–2.05)	0.489	43 (6.6)	1.08 (0.72–1.63)	0.709	16 (10.0)	1.65 (0.92–2.97)	0.094

Categorical variables are reported as counts and percentages. Risk estimates are hazard ratios for overall, 30-day and 5-year mortality and odds ratios for the other end-points.

Abbreviations: AF, atrial fibrillation; CI, confidence interval; eGFR, estimated glomerular filtration rate; STS, Society of Thoracic Surgery; TIA, transient ischemic attack.

*Variables considered in the adjusted analyses. Overall mortality: age, sex, third generation prosthesis, anemia, eGFR <30 mL/min/1.73m^2^, diabetes, pulmonary disease, severe frailty, active malignancy, NYHA4 symptoms, coronary artery disease, acute heart failure <90 days, transapical access, left ventricle ejection fraction <50%, extracardiac arteriopathy, combined aortic stenosis and regurgitation, length of hospital stay. 30-day mortality: third generation prosthesis, extracardiac arteriopathy, recent myocardial infarction, acute heart failure <90 days, NYHA4 symptoms, transapical access, eGFR <30 mL/min/1.73m^2^, STS score, recent aortic valve balloon valvuloplasty, length of hospital stay. 5-year mortality: age, sex, third generation prosthesis, anemia, eGFR <30 mL/min/1.73m^2^, diabetes, pulmonary disease, severe frailty, active malignancy, NYHA4 symptoms, coronary artery disease, acute heart failure <90 days, transapical access, left ventricle ejection fraction <50%, extracardiac arteriopathy, combined aortic stenosis and regurgitation, length of hospital stay. Early stroke: recent aortic valve balloon valvuloplasty. Late stroke: sex, third generation prosthesis, anemia, eGFR <30 mL/min/1.73m^2^, history or stroke or TIA, previous myocardial infarction, extracardiac arteriopathy, transapical access. Early or late stroke: sex, third generation prosthesis, anemia, eGFR <30 mL/min/1.73m^2^, history of stroke or TIA, previous myocardial infarction, extracardiac arteriopathy, transapical access.

Among the patients with no previous history of AF before the TAVR procedure, the independent predictors of new-onset AF were critical preoperative state (odds ratio (OR: 2.77; 95% CI: 1.20–6.43; p = 0.017), transapical access (OR: 2.63; 95% CI: 1.13–6.10; p = 0.025) and older than third generation prosthesis (OR: 1.57; 95% CI: 1.06–2.32; p = 0.025). Compared to patients who received a third generation TAVR prosthesis, moderate to severe paravalvular regurgitation was more common (1.9% vs. 7.2%, p<0.001) and operative risk higher as assessed by the STS score (3.23 (2.35–4.57) vs. 4.20 (2.99–6.20), p<0.001) or EuroSCORE II (3.84 (2.43–6.39) vs. 6.20 (3.58–11.10), p<0.001) in patients who received an older generation prosthesis. However, older than third generation prosthesis remained a predictor of new-onset AF even after forcing moderate to severe paravalvular regurgitation (p = 0.039), STS score (p = 0.034) or EuroSCORE II (p = 0.027) into the multiple logistic regression model. Hospital stay was longer in patients with new-onset AF (7 (4–12) days) compared to those with no AF (4 (2–6) days), paroxysmal AF (5 (3–7) days) or non-paroxysmal AF (5 (3–7) days) (p<0.001).

The antithrombotic therapies of the patients with AF at discharge are described in Table 3. In comparison with patients receiving any anticoagulant alone at the time of discharge, those patients receiving no antithrombotic therapy had a greater hazard of all-cause mortality during the follow-up, while no differences were observed relative to the other antithrombotic therapies. No differences in the incidence of late stroke was observed between any of the different antithrombotic combinations or lack thereof.

**Table 3:** Antithrombotic therapy among patients with atrial fibrillation at the time of discharge from the operative center.

Antithrombotic therapy		Overall mortality		Late stroke	
	n = 1092	Adjusted HR (95% CI)	P value	Adjusted OR (95% CI)	P value
AC	706 (64.7)	Reference	Reference	Reference	Reference
AC and SAPT	212 (19.4)	0.96 (0.75–1.22)	0.7330	0.97 (0.47–2.00)	0.942
AC and DAPT	41 (3.8)	0.78 (0.47–1.31)	0.353	0.36 (0.05–2.77)	0.328
DAPT	65 (6.0)	0.89 (0.60–1.32)	0.556	0.74 (0.21–2.57)	0.683
SAPT	26 (2.4)	0.81 (0.42–1.54)	0.513	2.40 (0.73–7.86)	0.148
None	42 (3.8)	7.15 (4.87–10.51)	<0.001	0.79 (0.18–3.47)	0.759

Categorical variables are reported as counts and percentages.

Abbreviations: AC, anticoagulant; CI, confidence interval; DAPT, double antiplatelet therapy; HR, hazard ratio; OR, odds ratio; SAPT, single antiplatelet therapy.

## Discussion

The current study documented that AF is a common condition among patients undergoing TAVR for severe AS with a prevalence of approximately 50%. A novel and relevant finding was that among patients with pre-existing AF undergoing TAVR, long-term mortality was significantly increased in those with a non-paroxysmal form of the arrhythmia, whereas paroxysmal AF had no effect on mortality. Secondly, new-onset AF was predicted by critical preoperative state, transapical access technique and use of older generation TAVR prostheses and was associated with both increased short and long-term mortality after TAVR. Incidence of stroke during short or long-term follow-up after TAVR was unaffected by the subtype of AF.

AF is a well-known predictor of mortality in the general population and in those with AS [16–18]. Research has also consistently demonstrated that pre-existing AF is associated with increased short and long-term mortality after TAVR [3, 5–10]. However, evidence has been lacking on whether the subtype of AF affects outcomes after TAVR. To date, there has been only one small cohort study, which reported that AF, regardless of subtype, was associated with increased mortality at one year of follow-up [10]. Several large population-based studies have demonstrated that in the general population paroxysmal AF confers no excess risk of mortality after adjusting for confounding factors [19–21], which is in line with the findings of the current work. The lack of association between paroxysmal AF and mortality most likely pertains to an earlier stage of cardiac degeneration compared to patients with non-paroxysmal AF.

The rate of new-onset AF in the current study was 7.2%, a finding in agreement with earlier studies [4–6]. Somewhat conflicting evidence on the effect of new-onset AF on mortality after TAVR has been presented in previous literature. A large meta-analysis by Sannino et al. did not find any effect of new-onset AF on mortality [7], whereas a more recent meta-analysis by Mojoli et al. demonstrated increased early and late mortality compared to sinus rhythm [8], which is in agreement with the current work. A recent cohort study reported increased mortality in patients with new-onset AF even in contrast to those with previous AF [9].

Transapical access was found to be an important predictor of new-onset AF as shown also by a previous pooled analysis [4]. The cause of excess new-onset AF associated with transapical TAVR may be attributed to inflammatory responses associated with epicardial and pericardial injury and ventilatory restriction, hyperadrenergic state and myocardial ischemia related to postoperative state after minithoracotomy [4, 22, 23]. However, it is possible that the increased rate of new-onset AF also relates to the greater burden of vascular disease in those selected for non-transfemoral access and not the access technique itself.

A novel and interesting finding of our study was that older generation TAVR prostheses were an independent predictor of new-onset AF. Older generations of TAVR prostheses are associated with a distinctly higher rate of paravalvular regurgitation [24, 25]. Significant paravalvular regurgitation, in turn, will lead to volume and pressure overload of the left heart, which could predispose to the emergence of new-onset AF, although this association was not observed in the current work. Additionally, newer generation prostheses induce less systemic inflammation compared to previous generations, which could also contribute to the fewer number of new-onset AF in this group of patients [26]. It may also be argued that the lower risk of new-onset AF associated with the use of newer generation prostheses could be explained by extending TAVR indication to patients at intermediate surgical risk during the more recent years. However, the evidence from the current adjusted analysis does not support this assumption.

AF is a major risk factor of thromboembolism and stroke, and stroke is in turn a common severe adverse event after TAVR [7, 27]. However, no association between any subtype of AF and stroke during the entire follow-up was observed in the current work. Indeed, more than a third of the strokes observed in the current study occurred during the index hospitalization, which implies a significant influence by procedural factors on early stroke incidence. Antithrombotic medication use is most likely an important factor behind the absence of association of any subtype of AF and early or late stroke. It is also possible that the generally low stroke number and the relatively short follow-up may be insufficient to draw any definitive conclusions. Previous research has demonstrated somewhat different results regarding the effect of AF on the incidence of stroke after TAVR. However, in a large meta-analysis by Sannino et al, pre-existing AF did not affect the incidence of early or late stroke after TAVR, whereas new-onset AF was associated with increased incidence of early, but not of late stroke [7]. Gargiuolo et al. replicated these findings regarding new-onset AF and stroke in a more recent meta-analysis [28]. Heterogeneous use of antithrombotic medications among patients undergoing TAVR probably contributes to the diverging outcomes between studies.

The optimal antithrombotic management after TAVR in patients with AF is unclear. Current guidelines recommend the use of a vitamin K antagonist either alone or in combination with an antiplatelet agent for 3 to 6 months [1, 2]. However, these recommendations are based mainly on expert consensus, and practices vary considerably. In the current work, no difference in stroke rates was observed between the antithrombotic regimes, whereas increased mortality in comparison to patients discharged with an anticoagulant medication alone was observed only in patients, who were discharged without any antithrombotic medication. This finding is not surprising, as these patients evidently represent a subset suffering from serious bleeding complications or too ill to withstand the use of necessary antithrombotic medications leading to significant predisposition for additional adverse events. The lack of association between different antithrombotic medications in AF patients and stroke may be related to the small number of patients discharged without an anticoagulant and the relatively short median duration follow-up. It is also possible that those without an anticoagulant at discharge started with one on a later date. Antiplatelet agents effectively prevent atherothrombotic strokes also in patients with AF, which may further affect the number of strokes in these patients. Indeed, as antithrombotic regimes were tailored according to individual risk assessment rather than a standardized protocol, it is not possible to draw any clear conclusions on the effect of different antithrombotic regimes on outcomes after TAVR based on the current results. Recently published results from the POPular TAVI trial suggest that oral anticoagulation alone is advantageous over oral anticoagulation in combination with clopidogrel in patients with AF undergoing TAVR, particularly due to fewer bleeding complications [29]. The ongoing randomized ATLANTIS trial comparing anticoagulant therapy with a vitamin K antagonist to apixaban among patients with a prior indication for oral anticoagulation therapy undergoing TAVR will likely further illuminate this pertinent question [30].

The present findings are of clinical importance because they suggest that non-paroxysmal AF, but not paroxysmal AF, is associated with structural changes of the atrial wall which have prognostic importance. Fibrosis of the atrial wall is an established trigger for AF and a recent study showed that focal left atrial fibrosis localized to the left inferior pulmonary vein antrum, the extent of left atrial fibrosis, and enlarged left atrial volume were independently associated with persistent atrial fibrillation rather than with paroxysmal atrial fibrillation in cardiac MRI [31, 32]. These findings may facilitate a better clinical stratification of the extent of cardiac damage in patients with AS [33].

There are some limitations to the current study that must be acknowledged. The main limitation is the retrospective design of the study with all its inherent challenges. New-onset AF was defined as being diagnosed during the index hospitalization for the TAVR procedure, the duration of which was variable, which may be seen as a limitation. By extending the period of post-operative detection, it is conceivable that the number of procedure-related new-onset AF cases could have been increased. Additionally, no routine electrocardiographic monitoring was performed prior to the TAVR procedure and it is therefore possible that some patients who were classified as having new-onset AF actually had undetected paroxysmal AF. Data on whether patients with new-onset AF had a paroxysmal or a non-paroxysmal form of the arrhythmia was not available, which is another limitation. A notable limitation is that the antithrombotic medication regimes between patients were not randomized and were rather based on individual risk assessment. Moreover, antithrombotic medications were recorded at admittance and discharge only, and possible regimen changes afterwards could not be accounted for. Lack of bleeding data is another limitation. On the other hand, these data represent a large unselected nationwide cohort of patients undergoing TAVR with a relatively lengthy follow-up strengthening the validity of the results. A particular strength of the study was the accuracy and completeness of the survival data.

In conclusion, among patients undergoing TAVR, pre-existing non-paroxysmal AF and new-onset AF are associated with increased mortality, whereas pre-existing paroxysmal AF is not. These findings suggest that non-paroxysmal AF rather than paroxysmal AF may be associated with structural cardiac damage which is of prognostic significance. Further large-scale studies are needed to confirm these observations.
